# Sociological study of religiosity in post-atheist Kazakhstan

**DOI:** 10.3389/fsoc.2025.1609030

**Published:** 2025-07-07

**Authors:** Aigul Zabirova, Natalya Seitakhmetova, Sholpan Zhandossova, Marhabbat Nurov

**Affiliations:** 1 Department of Public Opinion Monitoring, Kazakhstan Institute for Strategic Studies Under the President of the Republic of Kazakhstan, Astana, Kazakhstan; 2 Department of Religion Studies, Institute of Philosophy, Political Science and Religious Studies, Committee of Science, Ministry of Science and Higher Education of the Republic of Kazakhstan (CS MSHE RK), Almaty, Kazakhstan; 3 Faculty of Humanities and Law, Higher School of International Relations and Diplomacy, Turan University, Almaty, Kazakhstan

**Keywords:** religion, religiosity, secular, Kazakhstan, Muslims

## Abstract

This paper explores a marked shift in the role and functions of religion in Kazakhstan, indicative of change in ideology and societal foundations. In explaining this shift, we highlight various elements that affect the religious landscape in Kazakhstan, with the most pronounced and recent trend being the Islamization of society. The subject of this study is the exploration a person's daily religious rituals, participation in religious activities, reading religious publications, and discussing religion with other people. Our analysis revealed that though many people identify themselves as religious, few participate in religious life daily. That is why we argue that Islam in Kazakhstan remains as a cultural and traditional value for many Muslims, rather than a vital component of their everyday life.

## 1 Introduction

There has been a marked shift in the role and functions of religion in Kazakhstan, indicative of change in ideology and societal foundations. In explaining this shift, we highlight various elements that affect the religious landscape in Kazakhstan, with the most pronounced and recent trend being the Islamization of society. Political scientists and security experts conceptualize Islamization of society through the lens of radical Islamism, which is perceived by both the local population and religious scholars as a phenomenon predominantly influenced by external forces. This concept, originating in America, addresses the phenomenon of “radical Islamism in the twenty-first century”. It aims to underscore the negative effects of the Islamic factor on global stability and the potential threats posed by a religious reconfiguration of the world (Yom, 2020).

Historians and cultural scientists define religious renaissance as the projection of religion's socio-cultural function, which Kazakhs obtained because of independence (Burova and Jamanbalayeva, 2023; Sultangaliyeva, 1999). Indeed, the Islamic renaissance in Kazakhstan had and continues to have cultural importance, which is closely linked to processes of Kazakh ethnic identity renewal. During the early stages of nation-building, the resurgence of national and cultural traditions coincided with a renaissance of Islam, which is now incorporated into the notion of religious identity (Zabirova, 2003).

Sociologists view current religious architectonics as the result of “failed” modernization, when some social groups in society face social difficulties and life instability, which are accompanied by an ideological crisis, loss of values, and existential crises produced by rapid societal changes. In fact, global ideologies provoke the return to traditional uncertainty or demand authoritarian solutions to crises, which in turn causes radical Islam to expand within the countries themselves (Rashad and Sharaf, 2017).

The examination of the rise of religiosity reveals that a significant proportion of research, both domestic and international, focuses on the Islamic renaissance and revival, as evidenced by the works of Omelicheva (2011), Malik (2019), Aydingun (2007), Junisbai et al. (2017), Zabirova (2003), and Laruelle (2007). Based on quantitative data, Burova and Jamanbalayeva (2023), Zabirova (2024), Junisbai et al. (2017), Seitakhmetova et al. (2024), and Buribayev et al. (2024) identify socio-demographic characteristics as the most essential in understanding religious behavior and preferences, as well as geographic and social disparities among believers.

In examining Islam through a historical lens, Omelicheva (2010, 2011) and Aydingun (2007) emphasize its contextual relevance within specific locality. Historically in Kazakhstan Islam displayed a hybrid character, distinguished by the interplay of Islamic and pre-Islamic traditions. This is evident in the relatively low engagement in daily religious practices, with a more pronounced focus on significant life rituals, including childbirth, initiation stages (such as the 40 days and circumcision), weddings, and funerals.

This paper enhances the current body literature on religiosity in a post-Soviet nation, focusing on the contemporary religiosity of Kazakhs via the lens of current religious theories. The theoretical section of paper examines the concept of religiosity through such theoretical ideas as secularity, the individualization of religion, the “market model” theory, and post-secularity. This article provides also an overview of religious studies in Kazakhstan.

The empirical section of the essay outlines the findings of a social investigation. This paper aims to examine the religious and secular perspectives of the Kazakhstani population. The subject of who is a believer and who is religious has been debated for decades, and it remains open. Believers are described here as individuals for whom religious orientation is essential but not decisive, whereas religious are those for whom religious orientation is secondary to other, non-religious ones.

The Committee of Religious Affairs of the Republic of Kazakhstan, part of the Ministry of Culture and Information of the Republic of Kazakhstan, monitors the population's religious status quarterly. The primary distributions of the surveys are available on the open network and can be found on the Ministry's website. This paper is grounded on the information collected by the committee mentioned earlier (MLGroup, 2024). These large-scale surveys enabled us to test our theories. Specifically, we examine the data derived from four quarterly surveys conducted exclusively in 2024.

The focus of the research was on individuals residing in the Republic of Kazakhstan, specifically those within the age range of 18–65 years. The approach to gathering data involves conducting a comprehensive survey of individuals aged 18–65 years via direct personal interviews conducted in person. The research employed a multi-stage stratified sampling method that incorporated quotas. Every survey encompassed a total of 1.500 participants, with n1 = 1.500, n2 = 1.500, n3 = 1.500, and n4 = 1.500. A total of 6.000 individuals were surveyed throughout the duration of the study (MLGroup, 2024). It is essential to mention that the authors of this paper did not have access to the SPSS file. Thus, our analysis based on the descriptive report provided by the “MLGroup”, a polling company, which was published on the government website.

## 2 Literature review

In sociology, religion is traditionally understood as a system of ideas and practices that instill in people a sense of respectful fear and belonging to the sacred. Durkheim (1997), the classic sociologist of religion, defined a religious person as someone who engages in everyday religious rituals that give him a sense of solidarity and connection to a group of believers. In the framework established by Durkheim (1997), religion can be understood as a structured set of rituals, with an individual identified as religious through their engagement in these practices.

However, as evidenced by the literature review, modern theories of religion have a unique understanding of religion, religiosity, and secularization. Initially, we shall define the definitions of “religion”, “religiosity”, and “secularization” articulating these three components within the context of various contemporary theories.

The term religion derives from the Latin word religare, which translates to “to bind”. A person experiences a connection with the Absolute (the most perfect reference point) as a person's ability to identify with his nature. All religions share a fundamental concern for the harmonious coexistence of humanity. The cultivation of religious beliefs and faith in a higher power may indeed facilitate the resolution of issues pertaining to peaceful coexistence. Religion cultivates a sense of faith and hope, fostering kindness, truthfulness, and love within an individual's spirit.

The secularization theory is a predominant religious theory in the twentieth century. This approach originates from the nineteenth century, drawing on the ideas of Durkheim (1997). Starting in the 1950s, the idea was systematically developed in the writings of Berger (1967), Wilson (1982), and Norris and Inglehart (2004). The central idea of secularization theory is that modernization and its subsequent processes such a democratization, rationality, and urbanization diminish the importance of religion within society. Modernization results in the reduced importance of religion, causing it to lose societal significance, status, adherents, and the capacity to convey its own worldview. Proponents of this perspective perceive religion as a social institution that diminishes in relevance as society advances. Followers of the secularization idea see religion as a norm that is losing significance and becoming less common (Habermas, 2008). The religious element is crucial in shaping both confessional and individual religious culture.

Later other discourses began to take shape in theory. One of them is individualization theory which emerged in the 1970s, with its principal concepts formulated by Luckmann (1967), Davie (2013), and Hervieu-Léger (2000). They believed that people's religious preferences remained consistent; they simply lost touch with religious institutions. The disconnection from religious institutions results in the privatization and individualization of religion, fostering the expansion of personal faith. Davie (2013) is well—known for her concept of “believing without belonging”, wherein individuals may hold belief in God without participating in religious institutions. Luckmann (1967) proposed the idea of the “invisible hand”, wherein individual belief exists independently of conventional institutions. Hervieu-Léger (2000) articulated the concept of an individual's autonomy in selecting both religious beliefs and the specific components of religion that they find most appealing. In the twenty-first century, Campbell and Tsuria (2021) and Campbell (2005) theories on the individualization and privatization of religion have evolved through her research on digital religiosity. His study examines the shifts religion experiences due to new media and the impact of digital technologies on faith and religious practices.

Post-secularity, as articulated by such sociologists as Habermas (2008), Taylor (2007), and Casanova (1994), suggests that the relevance of religion persists amidst modernization, with religion continuing to fulfill a crucial role in the public sphere. Taylor (2007) examined the variety of religious and secular concepts present in contemporary society. Casanova (1994) discusses the emergence of a new phenomenon termed “de-secularization”, which refers to the resurgence of religious practices. He stated that secularization manifests differently across various societies, each exhibiting its unique characteristics. Various forms of secularization, whether historical or dialectical, may evolve asymmetrically, resulting in both the decline of religion and its revival (Taylor, 2007; Casanova, 1994).

Thus, according to the theory of secularization, religion gradually loses its social functions, the significance decreases; within the framework of the theory of individualization, religion becomes a personal, private matter of the individual; according to the theory of the “market model”, religion enters into competition with other social institutions and adapts to modern, post-modernization conditions; and within the framework of the theory of post-secularity, there is a revival of religion in new forms, reflecting a desire for a balance between post-secular and religious values. We argue that sociological theories of religion examined here complement one another in our analysis of re-Islamization in Kazakhstan. The idea is that during the Soviet era, the state vigorously secularized society; in the post-Soviet era, religion is undergoing a revival characterized by individualization, digitalization, a “market model” and post-secularity.

What concepts and theories are employed in the field of religious studies in Kazakhstan? Laruelle (2007) discusses “bourgeois Islam”, whereas Omelicheva (2010, 2011) observes features of commercialization and individualization of religion. Malik (2019), drawing on the concepts of Casanova (1994) regarding the “asymmetric development” of historical and dialectical interpretations of secularization, discusses a paradigm shift from secularization to re-Islamization in the Islamic world during the last quarter of the twentieth century. Malik (2019) analyzes the institutionalization of religion through the case study in Kazakhstan.

The research conducted by Burova and Jamanbalayeva (2023) combines state statistical data and findings from extensive surveys conducted in 2013, 2018, 2020, 2021, and 2022. Researchers describe the expansion of religious identity and its growing significance for the people of Kazakhstan. They highlight a specific diffusion of Kazakhstan's religious identity, indicating conflicting goals for both modernization and archaization in the perception of religious institutions.

Junisbai et al. (2017) conducted comparative studies in Kazakhstan and Kyrgyzstan in 2007 and 2012. The researchers proposed a typology of religiosity based on religious beliefs and religious practices. The survey results showed an increase in religious beliefs in both countries, in particular, people began to believe in Sharia law more, although the number of believers in Kazakhstan are significantly lower than in Kyrgyzstan. As for religious practices, the number of believers observing religious rituals grew only in Kyrgyzstan. In Kazakhstan in 2012, this number, on the contrary, decreased compared to 2007. Using logistic regressions, the researchers revealed the statistical significance of gender and ethnic factors in the formation of religious attitudes. The region of residence turned out to be an equally important factor in the formation of religious identity; it turned out that in both Kazakhstan and Kyrgyzstan, the population living on the South are more religious. Authors define the southern and western regions of Kazakhstan as more conservative, experiencing a strong influence of religious institutions in the process of socialization. While the northern region in Kazakhstan is more Russified, less involved in religious Islamic beliefs.

Comparative data on two countries, Kazakhstan and Turkey, is provided by Zabirova (2024) in her article “Religiosity in Kazakhstan: Values and Meanings”. The researcher compares the meaning of religiosity among Kazakhs and Turks, using representative data from the World Values Survey, she offers a typology of believers; where Turks are defined as practicing (devout or practicing Muslim), and Kazakhs as nominal (nominal or non-practicing Muslim) Muslims.

Omelicheva (2011) argues that the Islam practiced by nomadic peoples initially differed from that of sedentary Uzbek and Tajik Muslims. The Islam practiced by nomadic peoples was more influenced by local law (Adat) and exhibited a weaker connection to Sharia practices. The tsarist and Soviet regimes contributed to the development of many forms of religiosity in the region. The tsarist administration implemented various governance models: in nomadic societies, where Islam was considered a minor component of pre-Islamic traditions, the Russian administration encouraged Russification and upheld local law (Adat); in sedentary societies, more aligned with authentic Islamic practices, governance was conducted according to Sharia. The author emphasizes that in Kazakhstan, as a historically nomadic society, Islam was not as firmly rooted as among sedentary peoples; here, Islam was closely intertwined with pre-Islamic beliefs that the Kazakhs already had, acquired a certain local contextual character, and was less strongly connected to canonical Islam's religious practices.

Turkish academic Aydingun (2007) emphasizes that the religion among Kazakhs originally emerged as a synthesis of Sunni Islam from the Hanafi school, connected with indigenous Sufi traditions and certain pre-Islamic customs. She argues that pre-Islamic traditions like Nauryz were assimilated into the religious culture, alongside Kurban Ait (Eid al-Fitr) and Oraza Ait (Eid al-Adha). The practices of circumcision and burial rituals, along with rituals linked to births and weddings, possessed a religious significance and were consistently upheld across Kazakhstan. Significant religious ceremonies, including weddings and funerals, profoundly influenced the existence of the nomadic populations in Kazakhstan; however, routine rituals, like the five daily prayers, failed to integrate into the broader spectrum of religious observance. An additional significant aspect is that, even during the secular Soviet era, religion remained an integral part of Kazakh life, manifesting in essential rituals such as weddings, funerals, circumcision ceremonies, and more.

Even though religion was only a small part of the pre-Soviet identity mosaic and was suppressed and oppressed during the Soviet period, Islam enjoyed a resurgence and revival in the post-Soviet era. However, while Kazakhs are widely identified as Muslims, daily religious practices were hardly incorporated into everyday life. The practice of Islam among the Kazakhs has always displayed distinctive characteristics, integrating and synthesizing a multitude of beliefs, concepts, and identities. Islam amalgamated pre-Islamic shamanistic, Zoroastrian, and Tengrian rituals during the pre-Soviet era; the secularization of public space in both the Soviet and post-Soviet periods was accompanied by a dichotomy, marked by an increase in religiosity alongside a significant degree of secularization. An essential aspect of comprehending the expansion of religiosity is in examining the dynamics between the state and Islam in Kazakhstan. Numerous scholars have highlighted the contradictory character of the perspective on Islam. Malik (2019) emphasizes a duality in the stance of the Kazakhstani government regarding religiosity; while it appears to endorse its growth, this phenomenon simultaneously engenders controversy and debate. The Islamic revival is frequently framed within a security context, perceived as a potential challenge to the secular aspirations of Kazakhstan. Yemelianova (2013) underlined the complex response of the Kazakh elite to the resurgence of Islam, suggesting that the administration of first President, N. Nazarbayev, symbolically embraced the Kazakh Sufi legacy and the Hanafi school of Sunni Islam as “traditional” expressions of faith among Kazakh nomads, viewing them as essential components of the nation-building endeavor. However, various members of the political elite have, in fact, embraced the more unconventional Salafi Islam while disregarding, neglecting, or even restricting the resurgence of Kazakh Sufism as a genuine local tradition.

The bifurcated view of religion held by state institutions is further emphasized by Omelicheva (2011), she notes that this perspective began to take shape during the Soviet era. The Soviet era was predominantly marked by the enforcement of secular ideologies and the suppression of believers and religious leaders; however, it also experienced phases of ease and collaboration between the state and religious institutions. For example, during the Second World War, in 1943, Stalin established the Spiritual Administration of Muslims of Central Asia and Kazakhstan. In the era characterized by stagnation, known as the Brezhnev period, there commenced a phase of ideological reconfiguration of official Islam, aimed at reconciling its principles with the founding principles of communism. In the Brezhnev period, a proposition was advanced suggesting that one could simultaneously embody the virtues of both a devout Muslim and a committed communist (Yom, 2020). According to Omelicheva (2011), in the Soviet Union, an attitude toward religion developed when official Islam established by the state was regarded as “correct” and other religious institutions that the state did not legitimate were considered as “incorrect”. Part of this mindset persists today, when the state only deals with “official” Islam and views other religious movements with suspicion. She also mentions “alarming” aspects, such as the portrayal of Islam as a threat to Kazakhstan, despite the republic's prevalence of “moderate” and apolitical manifestations of Islam.

## 3 Research methods

This study examines religiosity and secular attitudes in Kazakhstan through the lens of sociological theories of religion. Several hypotheses were formulated regarding the religious landscape in Kazakhstan:

1. Islamic religiosity in Kazakhstan is characterized by hybridity, predominantly serving a cultural rather than quotidian function.
2. Kazakhstani society maintains a secular character despite increasing religious identification.
3. Religious institutions exert limited influence on individual decision-making compared to family and social networks.
4. Religious practices are undergoing digital transformation, with online platforms supplanting traditional modes of religious socialization.
5. Generational differences in religious engagement remain minimal despite overall increases in religious identification.
6. Government policy toward religion reflects an instrumental approach that incorporates traditional Islam into national identity while restricting autonomous religious movements.

### 3.1 Data source and limitations

This study utilizes secondary data from the Analytical Report “Evaluation of state policy of religion by Kazakhstani's population” conducted by “MLGroup” in Astana, Kazakhstan in 2024. This report is publicly available through the Committee of Religious Affairs of the Republic of Kazakhstan, a division of the Ministry of Culture and Information (https://www.gov.kz/memleket/entities/din/projects/1?lang=en).

It is important to acknowledge the significant methodological limitations inherent in our approach. Our analysis relies exclusively on the aggregated statistical distributions and pre-calculated metrics provided in the published analytical report rather than raw data files. This constraint was imposed by data availability, as the Committee does not release the underlying datasets (e.g., SPSS files) to external researchers. Consequently, our analytical capabilities were restricted to interpreting the descriptive statistics provided in the report, without the ability to perform additional data transformations, create new composite variables, or conduct more sophisticated statistical analyses.

The reliance on secondary, pre-aggregated data introduces several potential sources of bias:

1. Selection bias in the variables chosen for reporting by the original analysts.
2. Limited ability to identify or control for confounding variables.
3. Inability to verify the data cleaning procedures or analytical decisions made by the primary researchers.
4. Potential government influence on data presentation, given the official nature of the report.

Despite these limitations, we believe these data represent the most comprehensive and methodologically sound information available on religiosity in contemporary Kazakhstan, given the systematic quarterly collection methodology and nationally representative sampling strategy employed.

### 3.2 Survey methodology

According to the report, the surveys were conducted through face-to-face interviews with individuals aged 18–65 who resided in Kazakhstan. A multi-stage stratified sample with quotas was employed in the polling.

The sampling process involved multiple stages:

1. Regional sampling based on the “city-village/city-suburb” principle across Kazakhstan's 20 administrative-territorial entities (17 regions and 3 cities of republican significance: Astana, Almaty, and Shymkent).
2. Random selection of micro districts and streets from mapped lists of specified settlements.
3. Selection of households through a four-step process in urban areas and a two-step process in rural areas, with a restriction of one respondent per household.
4. Selection of respondents based on quotas for gender, age (18–29 years, 30–45 years, 46–65 years), and ethnicity (Kazakhs, Russians, other ethnicities).

The distribution of respondents by gender, age, and ethnicity was determined based on statistics from the National Office of Statistics for each region or city of republican significance. A total of 6,000 individuals were surveyed across four quarterly assessments in 2024, with 1,500 participants in each assessment (refer to Table 1).

**Table 1:** Distribution of population aged 18–65 years by types of settlement.

**Regions**	**Population aged 18–65, people**	**%**	**Number of respondents (per quarter), persons**	**Total number of respondents, people**
Republic of Kazakhstan	11,508,280	100.0	1,500	6,000
Akmola region	483,644	4.2	63	252
Aktobe region	539,479	4.7	70	280
Almaty region	861,261	7.5	112	448
Atyrau region	386,710	3.4	50	200
West Kazakhstan region	413,553	3.6	54	216
Zhambyl region	670,723	5.8	87	348
Karaganda region	694,518	6.0	91	364
Kostanay region	526,971	4.6	69	276
Kyzylorda region	456,463	4.0	59	236
Mangistau region	411,086	3.6	54	216
Pavlodar region	467,611	4.1	61	244
North Kazakhstan region	336,831	2.9	44	176
Turkestan region	1,099,384	9.6	143	572
East Kazakhstan region	446,456	3.9	58	232
Astana city	839,718	7.3	109	436
Almaty city	1,337,488	11.6	174	696
Shymkent city	652,297	5.7	85	340
Abay region	359,493	3.1	47	188
Zhetisu region	395,790	3.4	52	208
Ulytau region	128,804	1.1	18	72

### 3.3 Sample demographics

The analysis in this paper is primarily based on the fourth poll of 1,500 respondents. In this survey, 41.6% (*n* = 624) were young people aged 18–35. Gender distribution showed 48.9% (*n* = 733) male respondents and 51.1% (*n* = 767) female respondents. Among young people aged 18–35, 50.6% (*n* = 316) were men and 49.4% (*n* = 308) were women.

Age distribution of all respondents (18–65) was: 41.6% aged 18–35, 33.1% aged 36–45, 19.1% aged 46–59, and 6.2% aged 60–65.

The ethnic composition was Kazakhs −70.2%, Russians −15.7%, and other ethnic groups −14.1%. Among other ethnic groups, the largest percentages were Uzbeks (3.8%), Ukrainians (1.9%), Uighurs (1.6%), and Tatars (1.6%).

Regarding education levels, 36.5% of respondents had higher education, 30.7% had secondary education, and 32.7% had vocational education. Among respondents aged 18 to 35, 38.3% had higher education, 30.9% had secondary education, and 30.6% had vocational education.

In terms of employment, the majority (68.7% overall, 68.3% of youth) were employed, while others were housewives (10.1% overall, 8.7% of youth), students not currently employed (4.1% overall, 9.3% of youth), employed pensioners (2.6%), individuals temporarily on leave (3.1% overall, 2.6% of youth), those not working due to disability (2.7% overall, 2.1% of youth), unemployed pensioners (2.6%), working students (1.5% overall, 3.7% of youth), individuals neither studying nor working but looking for work (1.5% overall, 2.6% of youth), and officially registered unemployed individuals (1.3%).

### 3.4 Analytical approach and limitations

Our analytical strategy was necessarily constrained by the secondary nature of the data. The approach involved:

1. **Critical interpretation of descriptive statistics:** We carefully analyzed the frequency distributions, percentages, and cross-tabulations provided in the analytical report.
2. **Comparative analysis:** Where possible, we examined differences between demographic subgroups (particularly age cohorts) to identify patterns relevant to our hypotheses.
3. **Triangulation with qualitative insights:** The report included limited qualitative data from open-ended questions, which we integrated into our analysis to provide context for quantitative findings.
4. **Theoretical framing:** We interpreted the available data through established sociological frameworks on secularization, religious hybridity, and state-religion relations.

Several important analytical limitations must be acknowledged:

1. **Inability to conduct inferential statistics:** Without access to raw data, we could not perform tests of statistical significance, regression analyses, or other multivariate techniques that would allow for more robust hypothesis testing. Consequently, our findings regarding hypothesized relationships remain tentative and descriptive rather than definitively demonstrated.
2. **Limited variable transformation:** We could not create composite measures or scales that might better capture complex constructs like “religiosity” or “secularism”, instead relying on the pre-defined variables presented in the report.
3. **Missing temporal analysis:** While the report draws from quarterly surveys, the published results primarily present amalgamated findings rather than tracking changes over time, limiting our ability to examine temporal patterns.
4. **Potential reporting bias:** As the report was commissioned by a government agency with an interest in religious policy, the selection and presentation of findings may reflect political considerations rather than purely scientific objectives.

Despite these substantial limitations, we maintain that careful analysis of this data provides valuable insights into the religious landscape of Kazakhstan, particularly given the scarcity of comprehensive, nationally representative studies on this topic. We have approached the interpretation conservatively, avoiding claims that would require more sophisticated statistical validation, and have qualified findings appropriately throughout our discussion.

### 3.5 Hypothesis testing approach

Given the limitations outlined above, our approach to hypothesis testing is necessarily exploratory rather than confirmatory. For each hypothesis:

1. We identify the relevant variables from the analytical report that correspond to the concepts in our hypothesis.
2. We examine the percentage distributions across these variables and relevant demographic factors.
3. We interpret these distributions considering our theoretical framework, while clearly acknowledging the absence of statistical significance testing.
4. We qualify our conclusions appropriately, presenting them as provisional insights that warrant further investigation with more robust methodological approaches.

This cautious analytical strategy aims to extract valuable insights from the available data while maintaining scientific integrity by explicitly acknowledging the study's methodological constraints.

## 4 Ethics statement

This research utilized publicly available secondary data and did not involve direct data collection by the authors. All data analyzed in this study was obtained from an open source—the official website of the Committee of Religious Affairs of the Republic of Kazakhstan (https://www.gov.kz/memleket/entities/din/projects/1?lang=en), a division of the Ministry of Culture and Information.

The analysis in this paper is based entirely on the primary distributions published in the Analytical Report “Evaluation of state policy of religion by Kazakhstani's population” conducted by “MLGroup” in Astana, Kazakhstan in 2024. This report presents aggregated and anonymized data from surveys conducted by professional polling companies commissioned by the government.

In accordance with the Law of the Republic of Kazakhstan “On the Protection of Historical Data”, the processing of this publicly available, anonymized data does not require obtaining additional informed consent. No individual respondent data was accessed or processed during this research, only aggregated statistical information.

The original surveys were conducted by professional polling companies that won competitive tender officially announced by the Committee on Religious Affairs. These companies, not the authors of this study, were responsible for the original data collection, including obtaining appropriate consent from participants according to Kazakhstani research standards and regulations.

## 5 Results

### 5.1 Level of religiosity

The subject of this paper is the dynamics of changes in religiosity among young people as a distinct social group, as well as the dynamics of changes in religious views among three major ethnic groups: Kazakhs, Russians, and others ethnic groups. Table 2 defines the changes in religious beliefs throughout 2024 year within the populace of Kazakhstan. The examination of the research findings from all four surveys conducted in 2024 revealed insignificant changes between four seasons. The degree of religiosity among the population, we define as high, experienced a modest increase over the year, rising from 85.6% of respondents in the initial survey to 86.8% in the fourth survey.

**Table 2:** Responses to the question: “Please tell me, do you consider yourself a believer?”.

**Scale**	**Period**	**Population**	**Youth 18–35 years old**	**Ethnic groups**		
				**Kazakhs**	**Russians**	**Other**
Yes, I am a practicing believer, I strictly observe all the rules and rituals	Q1	19.1	18.1	22.1	9.8	13.7
	Q2	18.3	17.0	21.2	9.7	13.9
	Q3	18.2	17.6	21.2	9.4	13.2
	Q4	24.1	21.5	27.2	13.9	20.6
Yes, I am a believer, but I limit myself to holidays and observing some religious rituals	Q1	66.5	68.1	66.9	64.7	67.0
	Q2	67.4	69.4	67.2	67.2	68.4
	Q3	68.3	70.0	67.1	71.5	70.3
	Q4	62.7	65.9	62.9	60.5	64.1
No, I am not a believer, but I do observe certain religious rituals	Q1	7.1	8.5	5.0	16.2	8.0
	Q2	8.2	9.5	6.6	15.5	8.1
	Q3	9.9	10.1	8.6	16.6	9.0
	Q4	9.2	9.5	7.0	18.9	9.1
No, I am not a believer and I do not observe religious rituals	Q1	3.5	2.7	2.8	6.4	3.8
	Q2	2.9	1.9	2.6	5.0	2.4
	Q3	1.9	0.8	1.9	2.1	2.4
	Q4	1.3	0.8	0.8	3.8	1.0
No, I am not a believer, but I am calm about the fact that others do it	Q1	0.7	0.2	0.7	0.4	0.9
	Q2	0.5	0.3	0.5	0.4	1.0
	Q3	0.3	0.0	0.1	0.0	1.4
	Q4	0.5	0.3	0.4	0.4	1.0
I am a convinced atheist and I don't understand religious people	Q1	1.6	1.0	1.3	2.1	2.4
	Q2	1.3	0.6	1.0	1.3	2.4
	Q3	0.6	0.2	0.6	0.4	0.5
	Q4	0.9	0.5	0.8	1.3	1.0
I am an agnostic, I don't know if there is a God.	Q1	0.5	0.0	0.3	0.0	2.4
	Q2	0.6	0.0	0.2	0.4	2.4
	Q3	0.3	0.2	0.1	0.0	1.9
	Q4	0.5	0.3	0.3	1.3	1.0
Other	Q1	0.0	0.0	0.0	0.0	0.0
	Q2	0.0	0.0	0.0	0.0	0.0
	Q3	0.0	0.0	0.0	0.0	0.0
	Q4	0.0	0.0	0.0	0.0	0.0
Can't say	Q1	0.9	1.4	0.9	0.4	1.9
	Q2	0.5	1.1	0.8	0.4	1.4
	Q3	0.5	0.4	0.4	0.0	1.9
	Q4	0.8	1.3	0.8	0.4	1.4

In the surveys two definitions of religiosity used: “Yes, I am a practicing believer, I strictly observe all the rules and rituals” and “Yes, I am a believer, but I limit myself to holidays and the observance of some religious rites”, these exactly two statements were suggested to Poll company by one of the authors of this paper. As data shows, there is a small hence considerable increase in the number of people who identify as practicing believers, meaning they follow all religious ceremonies. In the first quarter, such believers constituted 19.1% of respondents, whereas by the fourth quarter, they increased to 24.1% of respondents. Those who declare themselves as believers, but merely observe religious holidays and some religious rites, have shown a minor decline in responders from 66.5% to 62.7% across all 2024 year.

Similar patterns observed among the youth: a rise in the proportion of practicing believers who adhere rigorously to all rituals (from 18.1 to 21.5% of respondents). The proportion of individuals who engage in only a limited number of religious practices has also experienced a slight decline, moving from 68.1 to 65.9% among respondents.

It is essential to note that the ethnic groups included in the survey demonstrate comparable trends. The proportion of practicing believers, or those who follow all rituals, increased by several percentage points among Kazakhs, from 22.1% in the first survey to 27.2% in the fourth, among Russians, from 9.8% in the first survey to 13.9% in the fourth, and among representatives of other ethnic groups, from 13.7% in the first survey to 20.6% in the fourth.

The proportion of individuals who engage in selective ritual observance experienced a modest decline throughout the analyzed timeframe: among Kazakhs, it decreased from 66.9% in the first survey to 62.9% in the final poll; among Russians, it fell from 64.7 to 60.5% in the fourth survey; and among members of other ethnic groups, it shifted from 67 to 64.1% in the concluding fourth survey. Although the number of practicing believers increased during the study period, while the proportion of believers who only observed some religious rituals fell, the overall proportion of believers who only observed some rituals was significantly higher than that of practicing believers.

These findings support our hypothesis that the number of Muslims who identify as believers will be high, while the proportion of practical believers who attend all rituals will be much lower than those who observe only some rituals.

The hypothesis suggesting that young individuals have higher levels of religiosity in comparison to other demographic groups lacks empirical evidence. Young people's religiosity is only slightly higher than the religiosity of the general population; 87.4% of respondents in the fourth survey among young people said they are religious, while 86.8% of respondents in the general population said the same.

Table 3 outlines the extent of engagement of the population, with particular emphasis on youth as a distinct social cohort, in religious rituals. This tables present information derived from all four surveys. First, the data indicate that many respondents engage in religious rituals. Specifically, the rituals surrounding childbirth, circumcision, matrimonial ceremonies, and funerary rites are observed with considerable frequency. The adherence to religious practices such as the five daily prayers and the observance of Sharia-compliant attire appears to be waning among the populace, particularly among the youth in Kazakhstan.

**Table 3:** Responses of Muslims to the question: “If you are a Muslim, what traditions and customs have you followed in your daily life over the past 12 months?”, %.

**Answer options**	**Measurement**	**Every day**	**Every week**	**Every month**	**2–3 Times in year**	**Once every few years**	**Never**	**Can't say**
Visiting a religious building (mosque, church, prayer house)	Q1	5.0	15.2	17.6	34.6	14.9	11.0	1.6
	Q2	5.5	14.7	17.1	34.0	16.4	10.7	1.5
	Q3	5.3	12.1	15.5	34.2	19.6	11.9	1.5
	Q4	6.2	14.5	17.5	33.8	16.1	10.4	1.5
Perform prayers	Q1	20.0	7.6	12.9	18.5	10.4	29.6	1.1
	Q2	19.0	8.3	13.1	13.1	12.0	28.5	1.1
	Q3	17.7	6.6	12.5	20.5	13.9	27.9	1.0
	Q4	19.3	8.2	13.3	18.5	11.8	27.9	1.0
Reading religious literature	Q1	4.9	4.6	11.2	25.9	14.1	36.8	2.4
	Q2	4.3	6.1	12.6	26.3	14.6	34.4	1.6
	Q3	5.4	4.2	12.4	26.4	21.7	28.0	1.9
	Q4	4.9	6.1	12.4	27.0	14.3	33.7	1.5
Watching religious TV channels	Q1	2.5	3.3	12.5	22.6	19.7	36.6	2.9
	Q2	2.3	4.2	13.5	23.9	21.7	32.5	2.0
	Q3	3.0	2.6	12.7	23.7	24.3	31.2	2.6
	Q4	3.3	4.1	13.2	24.3	21.3	31.9	1.9
Visiting religious websites	Q1	3.3	2.9	8.7	16.8	18.9	45.1	4.3
	Q2	3.0	4.2	10.1	17.7	21.6	41.0	2.4
	Q3	4.2	2.5	10.1	18.6	23.3	37.6	3.5
	Q4	3.8	4.2	9.9	18.1	21.3	40.4	2.4
Talking to relatives about religion	Q1	4.6	3.8	12.0	33.9	15.1	28.6	2.1
	Q2	4.0	4.0	12.1	32.5	18.1	27.9	1.3
	Q3	5.0	3.3	12.7	34.0	18.5	24.6	1.9
	Q4	4.3	4.0	12.2	33.0	17.9	27.3	1.3
Talking with friends and fellow believers about religion	Q1	3.7	3.2	14.8	29.1	18.3	27.2	3.8
	Q2	3.9	3.9	14.7	28.4	20.9	25.5	2.5
	Q3	4.1	2.9	15.5	29.1	21.8	23.2	3.4
	Q4	4.2	3.9	14.8	29.0	20.6	25.0	2.4
Talking to the clergy (imam, priest, pastor, rabbi)	Q1	1.6	3.6	7.4	29.2	20.2	36.4	1.6
	Q2	1.8	4.0	8.1	28.1	21.7	34.7	1.5
	Q3	2.1	3.1	8.2	30.1	22.1	33.1	1.3
	Q4	2.8	4.0	7.9	28.1	21.7	34.1	1.5

However, population holds religious beliefs, particularly in Allah, the Koran, and angels. The data substantiate the initial hypothesis regarding commitment, indicating that the level of religious beliefs is considerable. Though, the predominant segment of Muslims in Kazakhstan aligns themselves with the religious rituals that were existing in the Kazakh steppe prior to 1917 revolution. We're talking about rituals related to birth, initiation, marriage, and burials. The research revealed the customs that Muslims frequently observe in their daily lives: “sundet (sunnat)—the rite of circumcision of boys” −80.0% of participants (youth 80.4%); “faith in books, the Koran” −76.5% (youth 74.3%); “faith in Allah” −76.2% of participants [compared to the initial survey (81.3%) reflecting a decrease of −5.1%], while youth −73.7% (first survey 82.2%); “azan—a ritual at the birth of a child” −73.4% of participants (third quarter 78.1%), youth −73.3%; “janazah-namaz—a rite over the body of the deceased” −70.4% (third quarter 78.9%; −8.5%), youth −71.5% (third quarter 80.1%; −8.6% of participants).

Data from the fourth poll indicate 41.8% of believers engaged in the five daily prayers, while among the youth, the number was 41.5%. Additionally, 46.7% adhered to Sharia-compliant attire, with 46.2% of the youth conforming to this standard. Thus, despite the novelty of the five-time prayer for the modern population, the data acquired show growth in these activities, implying that over half of the country's Muslims began to do namaz (five times prayer).

Throughout the analyzed period, commitment to five daily prayers rose from 27.7% in the initial survey to 41.8% in the fourth survey across the population of Kazakhstan; among the youth, it increased from 26.5% in the first survey to 41.5% in the fourth survey. Sure, this represents a substantial increase.

Wearing clothing in accordance with Muslim rules. During the pre-revolutionary era, Kazakh women wore headscarves, however their faces, in contrast to other Central Asian inhabitants, remained uncovered. Dresses were traditionally Kazakh; historically, there was no distinct Muslim attire for Kazakh men and women. Secondly, the USSR actively discouraged Kazakh women from wearing native dress, particularly in the early years. One significant aspect contributing to the achievement of Soviet modernization in Central Asia was the project named “Liberation of Women of the East”. Currently, there is a notable rise in the number of individuals, both women and men, who choose to follow Muslim dress codes in Kazakhstan. Wearing Sharia-compliant clothes has increased among Kazakhstan citizens from 31.8% in the first survey to 46.7% in the latest survey, as well as among young people from 30% in the first survey to 46.2% in the latest survey. There are no distinctions in preferences for Muslim attire based on settlement type; among those who observe Sharia law in their dress choices, a marginally higher proportion are rural residents compared to urban ones.

Even though survey statistics show an increase in the practice of everyday religious rituals, they are still less popular among Kazakhstani believers than the “big”, traditional Kazakh rituals that accompany the most important events in a person's life. In terms of the hypothesis that young people are more religious, the poll results do not support this. Young people in Kazakhstan exhibit similar religious dynamics to other age groups.

The next hypothesis is that religion in Kazakhstan has yet to become an integral component of believers' daily lives. Respondents who consider themselves adherents of faith seldom engage with religious institutions, partake in prayers, or discuss matters of spirituality with their families, fellow practitioners, or religious leaders. Empirical evidence indicates that the proportion of individuals who engage in daily and weekly prayer is less than that of those who do not pray at all (27.5% of respondents). A significant portion of respondents, specifically 33.8%, report visiting religious organizations such as mosques, churches, and prayer houses only 2–3 times annually.

It is worth noting that many respondents (33.7%) never read religious literature, never watch religious television (31.9%), never visits religious websites (40.4%), or never speaks with clergy (34.1%). Regarding discussions about religion with family and friends, a significant portion of respondents (33%) indicated that they engage in such conversations with only relatives 2–3 times annually, while (29% of respondents) similarly converse on religious matters with friends during the same frequency. The findings support the study's third hypothesis, that essential religious activities are not fully integrated into daily life (Table 4).

**Table 4:** Responses to the question: “How often do you perform the following actions?”, %.

**Answer options**	**Measurement**	**Every day**	**Every week**	**Every month**	**2–3 Times in year**	**Once every few years**	**Never**	**Can't say**
Visiting a religious building (mosque, church, prayer house)	Q1	5.0	15.2	17.6	34.6	14.9	11.0	1.6
	Q2	5.5	14.7	17.1	34.0	16.4	10.7	1.5
	Q3	5.3	12.1	15.5	34.2	19.6	11.9	1.5
	Q4	6.2	14.5	17.5	33.8	16.1	10.4	1.5
Perform prayers	Q1	20.0	7.6	12.9	18.5	10.4	29.6	1.1
	Q2	19.0	8.3	13.1	13.1	12.0	28.5	1.1
	Q3	17.7	6.6	12.5	20.5	13.9	27.9	1.0
	Q4	19.3	8.2	13.3	18.5	11.8	27.9	1.0
Reading religious literature	Q1	4.9	4.6	11.2	25.9	14.1	36.8	2.4
	Q2	4.3	6.1	12.6	26.3	14.6	34.4	1.6
	Q3	5.4	4.2	12.4	26.4	21.7	28.0	1.9
	Q4	4.9	6.1	12.4	27.0	14.3	33.7	1.5
Watching religious TV channels	Q1	2.5	3.3	12.5	22.6	19.7	36.6	2.9
	Q2	2.3	4.2	13.5	23.9	21.7	32.5	2.0
	Q3	3.0	2.6	12.7	23.7	24.3	31.2	2.6
	Q4	3.3	4.1	13.2	24.3	21.3	31.9	1.9
Visiting religious websites	Q1	3.3	2.9	8.7	16.8	18.9	45.1	4.3
	Q2	3.0	4.2	10.1	17.7	21.6	41.0	2.4
	Q3	4.2	2.5	10.1	18.6	23.3	37.6	3.5
	Q4	3.8	4.2	9.9	18.1	21.3	40.4	2.4
Talking to relatives about religion	Q1	4.6	3.8	12.0	33.9	15.1	28.6	2.1
	Q2	4.0	4.0	12.1	32.5	18.1	27.9	1.3
	Q3	5.0	3.3	12.7	34.0	18.5	24.6	1.9
	Q4	4.3	4.0	12.2	33.0	17.9	27.3	1.3
Talking with friends and fellow believers about religion	Q1	3.7	3.2	14.8	29.1	18.3	27.2	3.8
	Q2	3.9	3.9	14.7	28.4	20.9	25.5	2.5
	Q3	4.1	2.9	15.5	29.1	21.8	23.2	3.4
	Q4	4.2	3.9	14.8	29.0	20.6	25.0	2.4
Talking to the clergy (imam, priest, pastor, rabbi)	Q1	1.6	3.6	7.4	29.2	20.2	36.4	1.6
	Q2	1.8	4.0	8.1	28.1	21.7	34.7	1.5
	Q3	2.1	3.1	8.2	30.1	22.1	33.1	1.3
	Q4	2.8	4.0	7.9	28.1	21.7	34.1	1.5

According to survey data, the population's religiosity remains stable, and most of the population continues to practice only those traditional rituals that associated with the most important life events, such as circumcision (80% of respondents), childbirth rituals (73.4% of respondents), and funeral rites (70.4% of respondents).

Such new religious norms as five times per day prayer and wearing Sharia-compliant clothing maintained to a lesser level. Although these religious norms are becoming less favored among believers in Kazakhstan, there is still a noticeable rise in these practices.

The role of religion in daily life in Kazakhstan. The proportion of respondents who pray daily or weekly (27.5%) is about like the proportion who never pray at all (27.9%). Approximately one-third of respondents in Kazakhstan (33.8%) attend mosques, churches, or prayer houses only 2–3 times annually. A significant portion of respondents reported that they do not engage with religious literature (33.7%), do not view religious television channels (31.9%), and do not access religious websites (40.4%). Most people only talk about religion with close friends and family (33%) and acquaintances (29%) a couple of times a year, and they hardly talk to clergy. One of our essential observations is that though many people identify themselves as religious, few participate in religious life daily. That is why, despite a high level of religious affiliation, we argue that Islam remains a cultural and traditional value for many Kazakhstan's Muslims, rather than a vital component of their everyday life, indicating similarities with Muslim communities in the Middle East.

The hypothesis suggests that young individuals tend to be more religious, as they are often the first to encounter various social changes. Consequently, the resurgence of religiosity observed in Kazakhstan is expected to primarily influence this demographic. The data collected to date do not support this assumption, as the religiosity of young individuals, along with their attitudes and behaviors, shows minimal variation compared to the broader population indicators.

### 5.2 The digitalization of religion

Evidently, if people are subjected to religious insults, this occurs more frequently online, through the consumption of old and new types of media. Thus, many respondents (19.3%) claimed that they encounter offensive content on the Internet, 16.2% reported facing religious insults while using social media platforms. 11.5% of respondents indicated that the media disseminate content that disparages their religion. Only 7.8% of respondents reported cases of personal insults or refusal by clergy to attend a religious building (mosque, church). The statistics reveal that, first and foremost, religious rights are violated in the media (Table 5).

**Table 5:** Responses to the question: “In what specific manner were your rights to freedom of religion infringed?”.

**Answers**	**Population**		**Youth 18–35 years old**	
	**Amount**	**%**	**Amount**	**%**
Public dissemination of materials offensive to my religion in the media	157	11.5	63	6.1
Offensive comments about my religion on social media	221	16.2	97	9.4
Posting offensive pictures to my religion on the Internet	264	19.3	111	10.8
Refusal of my visit to a religious building (mosque, church) by the clergy	107	7.8	42	4.1

As we noted above, in the 2000s, a new idea in religious studies emerged, known as Digital Religion. Sociologist Heidi Campbell was among the pioneers in examining how the evolution and impact of digital culture alter conventional religious practices and the operations of religious organizations (Campbell and Tsuria, 2021). The poll statistics indicate that for Kazakhstanis, the primary sources of information about religion are the Internet and social media. Considering this, 40% of people say they obtain their news through social media, and 29% say they get their news through instant messaging. All of this indicates that traditional media are replacing by the digital one. Currently we do observe novel habits among believers, which are transitioning to a digital format. Thus, 21.1% of respondents get their knowledge from YouTube channels, and 18.6% from news websites, indicating the popularity of video content and news sites on the Internet in Kazakhstan.

Traditional types of disseminating religious information are losing popularity: if 18.6% of respondents get their information from news websites, only 11.8% go to houses of worship. The respondents recognize the impact of the Internet and social networks on religion and contemporary life. In response to the question: “What elements do you believe contribute to the increasing number of young individuals embracing religion?” 34.7% of respondents indicated “the influence of propaganda on the Internet” as the most significant factor. Other explanations were “imitation of peers inherent in young people” (27.0% of respondents) and “fashionable hobby” (18.7%). The population attributes the influence of youth to “family upbringing” to the least extent, at 14.2%. Consequently, it may be inferred that the institution of the family is diminishing as a facilitator of religious socialization, while the influence of the Internet is growing (Table 6).

**Table 6:** Responses to the question: “What factors do you believe contribute to the increasing number of young individuals embracing religion?”, %.

**Answers**	**Measurement**	**Population**	**Youth 18–35 years old**
The influence of foreign preachers	Q1	20.9	8.5
	Q2	21.8	8.8
	Q3	19.0	9.2
	Q4	21.8	9.4
The impact of internet propaganda	Q1	36.0	15.1
	Q2	35.7	15.2
	Q3	29.2	14.7
	Q4	34.7	16.1
Return to sincere faith, religion	Q1	18.7	8.5
	Q2	18.8	8.6
	Q3	16.0	8.9
	Q4	18.5	9.2
Restoration of folk traditions	Q1	17.7	8.7
	Q2	18.1	8.9
	Q3	15.1	8.6
	Q4	17.4	9.2
Filling the spiritual void	Q1	15.9	6.0
	Q2	15.8	6.3
	Q3	13.8	6.3
	Q4	15.2	6.6
Fashion craze	Q1	21.4	8.9
	Q2	21.4	9.1
	Q3	27.5	12.9
	Q4	18.7	8.6
Imitation of peers, which is typical for young people	Q1	29.0	14.0
	Q2	29.0	13.9
	Q3	22.7	12.9
	Q4	27.0	14.3
Family education	Q1	33.9	17.2
	Q2	34.0	17.2
	Q3	25.3	15.9
	Q4	14.2	16.2
Influence of the environment (friends, acquaintances)	Q1	31.1	13.1
	Q2	28.8	12.0
	Q3	20.9	15.5
	Q4	23.2	10.4
Other	Q1	0.2	0.0
	Q2	0.3	0.0
	Q3	0.1	0.0
	Q4	0.3	0.0
Total answers	Q1	224.8	205.4
	Q2	223.7	206.4
	Q3	189.7	186.2
	Q4	205.9	281.9

Our statistics indicate that digitalization has already transformed the religious behaviors of the population in country. The Internet and social networks have emerged as the primary sources of religious knowledge. 40% of respondents get information from social networks; 29% of respondents use instant messengers; 20% of respondents view YouTube videos; and 18.6% of respondents visit online news sites. Simultaneously, 11.8% of respondents acquire religious knowledge in houses of worship. In addition, respondents frequently encounter offensive religious content in the online arena. The digitalization of the religion is that most respondents identified the Internet as the primary source of religious propaganda, whereas family ranked last as a source of religious socialization. The impact of access to digital resources on the evolving role of religion in Kazakhstan is evident; religion is increasingly adopting a hybrid nature, merging digital and traditional elements.

### 5.3 Government and religious discourse

As Omelicheva noted current state attitudes toward religion in Kazakhstan originated from the USSR, where Islam was categorized into “official” and “unofficial” or “correct” and “incorrect”. Her idea is valid, as post-independence Kazakhstan embarked on a trajectory of institutionalizing religion, recognizing official Islam as integral to national identity, while perceiving uncontrolled religious movements as a potential threat, thereby relegating religion to the framework of securitization.

In this sense is essential to look at the Law on Religious Activity and Religious Associations, which was enacted from October 11, 2011. The Law governs the operations of both recognized and unofficial religious organizations. In Kazakhstan, traditional Islam is predominantly Sunni, namely following the Hanafi madhhab, which is adhered to by many Muslims. In addition to Sunnism, there are some various sects of Islam, including Salafism and Wahhabism. As Aydingun indicated Wahhabism predominantly exerts influence in the Fergana Valley of Uzbekistan, with a minimal presence in certain regions of Kazakhstan. Seems her assertion that Wahhabism exerts greater influence inside the concentrated Uzbek community than among ethnic Kazakhs is still valid.

The degree of religiosity is different across country. The South displays higher level of religiosity, particularly in the Turkestan region where 72% of individuals identify themselves as believers, and in the city of Shymkent, where this figure rises to 77.6% among respondents. Western Kazakhstan is rapidly emerging as a second region with an increase in religiosity. In the Atyrau region, 46% of respondents identified as religious; in the Aktobe region, this figure stands at 38.6%; and in the Mangistau region, 37% of individuals consider themselves as believers. Even though Kazakhstan's south and west are more religious, data show that population in these two regions are less familiar with the “On religious activity and religious associations” law, this law governs the country's religious domain. The knowledge about the law in these locations is quite low, implying that the populace has limited legal literacy on religious issues. Considering the fact these regions are home to a significant population of various ethnic groups, specifically the Uzbeks on the south and the Karakalpaks in Mangistau.

The population's awareness and appraisal of state religion policy are dependent on transparency, information accessibility, and the effectiveness of the implemented measures. Interfaith harmony, safeguarding against extremism, and guaranteeing freedom of religion are the pillars upon which the religious policy of Kazakhstan rests. Kazakhstan is actively engaged in informing its population and implements its state policy in the religious domain through official channels. According to the fourth poll, many Kazakhstanis (88.6% of respondents) and young people (89.2%) favor official religious policy. A total of 41.0% of respondents from the population express full support for the state policy in the religious sphere, while 47.6% indicate a tendency to support it. A total of 42.1% of the youth fully support it, while 47.1% of respondents express a tendency to support it (Table 7).

**Table 7:** Responses to the question: “Do you support or oppose the state policy in the religious sphere?”, %.

**Do you support or not the state policy in the religious sphere?**	**Population**	**Youth 18–35 age**
I completely agree	41%	42.1%
I rather agree	47.6%	47.1%
Rather disagree	6.8%	6.4%
I completely disagree	2.7%	3%
I can't answer	1.9%	1.3%

Thus, we do define state religious policy as instrumental, particularly in relation to Islam, because Islam, sponsored by the state, is an integral element of national identity, however the state minimizes the influence of foreign religious movements.

In Kazakhstan, traditional Islam is symbolized by Sunni Islam of the Hanafi madhhab, to which many Muslim adherents. Non-Sunni movements do, however, emerge, particularly in locations with a high level of religiosity and the existence of multiple ethnic groups practicing different kinds of Islam. As it had been stated previously, public policy toward Islam can create a double impression, because Islam is both a recognized element of national culture and a potential source of threat. Despite this, many Kazakhstan's inhabitants support the government's religious policies.

### 5.4 Being religious vs. being secular

In classical sociology, secularization refers to the process of lowering the significance of religion in people's consciousness and social life; the shift from a society governed primarily by religious tradition to a secular model of social order based on rational (non-religious) principles. Secularization also refers to official policies that aim to reduce religion's influence and role, such as secularizing education (Norris and Inglehart, 2004; Wilson, 1982). Religion is seen in very different light in such contemporary sociological theories of religion as secularization theory, the commercialization of religion, the “market model” of religion, and post-secularity theory, the process of secularization. According to secularization hypothesis, religion in modern society is giving way to science, politics, and economics as a natural process of human progress. In severe instances, religion can completely disappear from society's life.

People reject old forms of religiosity within the framework of the privatization of religion idea, but this does not imply that religion has vanished, but rather that it has evolved. According to this theory, secularity signifies a divergence from institutional religion rather than from spirituality (faith). In the context of market model theory, religiosity does not vanish; instead, it is restructured in accordance with market principles. According to this theory, secularity entails increased religious choice and competition. According to post-secularity theory, a secular society does not have to be anti-religious; people in such cultures can blend secular and religious meanings; based on our findings, Kazakhstanis' religious convictions do not cancel out their secular culture.

In recent decades, there has been a notable transformation in the perspectives surrounding religiosity and secularity in Kazakhstan. The Soviet era was predominantly marked by the expansion of secular perspectives and the suppression of religious practices. During the post-Soviet era, the concepts of religious individualization, digitalization, and post-secularity have been partially realized within the nation. From our perspective, population in Kazakhstan is simultaneously religious and secular. Country promotes secular ideals, which include the separation of religious institutions from the state as well as the state's neutrality in matters of faith. The primary objective of secular principles is to guarantee the equal standing of all individuals, irrespective of their religious convictions, while safeguarding their right to freedom of conscience. The concepts underlying a secular state include the separation of the state and religious groups, the preservation of people's religious freedoms, the balance of all religions in the state, and the provision of secular education to citizens. As a result, these principles ensure the adherence to fundamental civil rights and freedoms as outlined in the nation's Constitution. This, in turn, indicates the formation of secular state.

In this study, the level of secularity is measured by two questions: who did the respondent turn to for support in a tough life situation and if they regard Kazakhstan to be a secular state. Table 8 contains data about support in a tough life situation. The primary distributions indicate that in challenging circumstances, individuals tend to seek assistance from family, relatives, or friends rather than from spiritual leaders or clergy.

**Table 8:** Responses to the question: “In the past 6 months, during challenging life circumstances, whom have you sought for guidance, assistance, or support?”, %.

**Over the past 6 months, in difficult life situations, who have you turned to for advice, help, support?**	**Population**	**Youth 18–35 age**
To parents, children	52.1%	36.20%
To relatives	42.2%	28%
To friends	31.1%	23%
To groups in messengers	2.4%	1.6%
To the clergy	4.8%	3.3%
To psychologists, crisis center workers	3.8%	2.2%
Other	6.5%	4.2%

Table 9 presents the data regarding the populace's perspective on the secular state. The data indicate that a significant majority of the population (84.5% of respondents) agrees with the statement that “In a secular state, freedom of conscience is guaranteed across all domains of life, encompassing religion, and that religion does not encroach upon state policy”. “What are your thoughts regarding Kazakhstan's status as a secular state?” Of course, attitudes toward a secular state are not static but rather dynamic, influenced by a variety of variables. Political and cultural transformations, alongside educational initiatives and public discourse, possess the capacity to shape and evolve these relationships over time.

**Table 9:** Responses to the question: “In a secular state, freedom of conscience is ensured in all spheres of life, including religion, and religion does not interfere with state policy. How do you feel about the fact that Kazakhstan is a secular state?”, %.

**How do you feel about the fact that Kazakhstan is a secular state?**	**Population**	**Youth 18–35 age**
I completely agree	48.2%	45.8%
I rather agree	36.3%	38%
Rather disagree	8.6%	9.6%
I completely disagree	1.6%	1.3%
I can't answer	5.3%	5.3%

Thus, Kazakhstan's population blends secular attitudes with a religious worldview; here, a person respects the state's secular values, but religiosity also plays an important role in the lives of some people. As indicators of the population's secular nature, we emphasize that the residents of the republic typically do not pursue assistance or guidance from spiritual leaders and clergy. Many respondents said they prefer to turn to family in difficult situations, while almost one-third said they prefer to turn to friends. A mere 4.8% of the nation's populace and 3.3% of its youth have turned to spiritual leaders and religious figures for assistance. In other words, religious institutions and communities have not evolved into population-supportive actors. Furthermore, despite the high level of religiosity, the absolute majority agreed that Kazakhstan should remain as a secular state. All of this suggests that Kazakhstan is developing a hybrid paradigm of secularity in which secular ideals prevail, yet religiosity is tolerated.

## 6 Discussion

The empirical findings presented in this study can be effectively interpreted through the lens of the various theoretical frameworks discussed in the literature review, particularly the secularization theory, individualization theory, market model, and post-secularity perspective. This integration provides a more comprehensive understanding of the religious dynamics in Kazakhstan.

### 6.1 Secularization theory and empirical evidence

Our data both support and challenge aspects of the secularization theory. While modernization has indeed led to a shift in religious practices, with many Kazakhstanis maintaining a secular outlook (84.5% supporting Kazakhstan as a secular state), there is no evidence of religion diminishing in significance overall. Rather than witnessing a decline in religiosity as predicted by classical secularization theory, we observe a high level of religious identification (86.8% of respondents identifying as believers by the fourth survey). This suggests that the linear progression toward secularization proposed by scholars like Berger (1967), Wilson (1982), and Norris and Inglehart (2004) is not fully applicable in the Kazakhstan context. Instead, we see what Casanova (1994) described as an “asymmetric development” of secularization, where religious revival coexists with secular values.

### 6.2 Individualization theory in practice

The individualization theory, as proposed by Luckmann (1967), Davie (2013), and Hervieu-Léger (2000), finds strong support in our empirical data. The significant gap between those who identify as believers (86.8%) and those who actively practice all religious rituals (24.1%) exemplifies Davie's concept of “believing without belonging”. Our findings reveal that many Kazakhstanis maintain religious beliefs while being selective about which practices they observe. The preference for traditional rituals such as circumcision (80%), childbirth ceremonies (73.4%), and funeral rites (70.4%), over daily prayers (41.8%) and Sharia-compliant attire (46.7%), illustrates Hervieu-Léger's notion of individual autonomy in selecting religious components that resonate most personally.

### 6.3 Market model application

The market model theory is evidenced in our data through the competition between traditional and newer religious practices and information sources. The increasing pluralization of religious information sources, with 40% of respondents obtaining religious knowledge from social networks, 29% from instant messaging, and 21.1% from YouTube channels, demonstrates how religion has entered a competitive marketplace of ideas. Religious institutions are adapting to these market conditions, as evidenced by the relatively low proportion (11.8%) of respondents who acquire religious knowledge in houses of worship compared to digital sources. This aligns with the market model's prediction that religion restructures itself according to market principles in contemporary societies, supporting Omelicheva's (2010, 2011) observations on the commercialization and individualization of religion in Kazakhstan.

### 6.4 Post-secularity in Kazakhstan

Our findings provide compelling evidence for post-secularity as theorized by Habermas (2008), Taylor (2007), and Casanova (1994). The data demonstrate that Kazakhstanis simultaneously hold both religious and secular values—a hallmark of post-secular societies. Despite high levels of religious identification, 84.5% of respondents support Kazakhstan's status as a secular state, and only 4.8% turn to religious leaders during difficult life circumstances. This indicates a post-secular balance where religion remains significant in the public sphere while secular principles are preserved. The revival of religious practices, particularly among ethnic Kazakhs (increase from 22.1 to 27.2% in practicing believers), alongside strong support for secular governance (88.6% supporting state religious policy), exemplifies what Taylor (2007) described as the variety of religious and secular concepts coexisting in contemporary society.

### 6.5 Digital religion and its theoretical implications

Campbell and Tsuria's (2021) and Campbell (2005) work on digital religiosity finds direct application in our empirical data. The predominance of digital sources for religious information and the identified role of the internet in religious socialization (34.7% citing “the influence of propaganda on the Internet” as the primary factor in young people embracing religion) support Campbell's theories on how digital technologies transform religious practices. This digital dimension adds nuance to the individualization theory by highlighting new mechanisms through which religious beliefs are personalized and privatized in contemporary Kazakhstan.

### 6.6 Hybrid religiosity: a theoretical integration

Our empirical findings suggest that no single theoretical framework fully captures the complexity of religious dynamics in Kazakhstan. Instead, we observe what might be termed “hybrid religiosity”—a phenomenon that integrates elements from multiple theoretical perspectives. This hybrid model exhibits:

1. **Selective secularization:** Certain aspects of social life remain secular (governance, education) while religion maintains significance in others (identity, rituals, personal belief).
2. **Individualized practice:** Following the individualization theory, Kazakhstanis create personalized religious expressions by selecting which rituals and beliefs to maintain.
3. **Market-driven adaptation:** Religious information and practices respond to competitive forces, particularly in digital spaces.
4. **Post-secular balance:** The simultaneous embrace of both religious identification and secular governance principles.
5. **Digital mediation:** As Campbell theorized, digital technologies fundamentally alter how religion is experienced, shared, and practiced.

This hybrid religiosity model helps explain the seemingly contradictory findings in our data: high levels of religious identification alongside strong support for secular principles; preference for traditional rituals while embracing new digital religious information sources; and increasing religious identification without corresponding increases in institutional religious participation.

The re-Islamization process in Kazakhstan thus represents not a simple return to pre-Soviet religious patterns, but rather the emergence of a distinctly modern, hybrid form of religiosity that responds to Kazakhstan's unique historical, cultural, and technological context. This supports Malik's (2019) analysis of the shift from secularization to re-Islamization in Kazakhstan, while adding nuance regarding the specific character of this religious revival.

## 7 Conclusion

According to presented data, many people in post-atheistic Kazakhstan continue to live in a secular culture. Yes, population identifies as believers, yet Islamic religion is only practiced by a small percentage. Our analysis revealed that most survey participants profess religion but do not prioritize religious norms and values in their daily lives. While there are some devout Muslims in Kazakhstan, they are a minority. However, believers in Kazakhstan are no longer a homogeneous community. That is why, in this study, the subject of investigation was a person's practice of conducting daily religious rites, participating in religious organization activities, reading religious publications, and discussing religion with other people. The measurements we took revealed two distinct groups with varying levels of religiosity and lifestyle. However, all these processes and phenomena are novel, marked by fluctuations, which necessitates sociological monitoring as a system of continuous observation of the dynamics of changes in the level of religiosity among the population.

The study revealed no violations of religious rights among the population and youth, suggesting a lack of substantial restrictions on religious freedoms. However, it is evident that everyday discrimination based on religion persists within the community, highlighting the necessity to explore rational strategies for its prevention.

Surveys data indicates that the population's level of religiosity remains elevated. Concurrently, a significant portion of the populace remains committed to the customary practices linked to the historical ceremonies of the Kazakhs, including circumcision, childbirth rituals, and funeral rites. The most essential religious rituals in classical Islam countries (meaning the Middle East as an Islamic citadel) are observed to a lesser level in Kazakhstan, the most important of which being five times per day prayer and Sharia-compliant clothing. To put it differently, religion plays a role in everyday life in Kazakhstan, albeit in a somewhat subdued manner. The proportion of those who pray daily or weekly is nearly like the proportion who do not pray at all. A significant majority of respondents attend mosques, churches, or prayer houses merely 2–3 times annually. Many participants do not engage with religious literature, do not watch religious television channels, nor do they explore religious websites. Many participants engage in discussions about religion with family and friends merely two to three times annually. There is a strong sense of religious identification, but few people take part in religious activities. Despite the high degree of religious affiliation, religion remains a cultural and traditional value for most Kazakhstan's population rather than a vital component of their daily lives.

The second hypothesis suggests that the increased religiosity among young individuals is a direct reflection of the social transformations and the resurgence of religious sentiment observed in Kazakhstan, suggesting that this phenomenon will manifest predominantly within the youth demographic. However, the data collected do not support this notion.

The advent of digitalization has significantly transformed religious practices in Kazakhstan. The Internet and social networks have emerged as the primary conduits for religious information, with individuals increasingly relying on platforms such as social networks, instant messaging applications, YouTube, and various news websites within the digital realm. At the same time, only 11.8% of respondents obtain religious information from places of worship. Respondents also confront obstacles in the online arena, such as offensive religious content. Another element contributing to the digitalization of the religious sphere is that many respondents identified the Internet as the primary source of religious propaganda, while the family's role as a source of religious socialization is declining. Consequently, one may infer that the religious landscape in Kazakhstan is evolving toward a hybrid character, integrating both digital and traditional elements.

The state adopts a pragmatic stance toward Islam, integrating it as a component of national identity while concurrently constraining the impact of autonomous religious movements. The state's approach to Islam presents a dual perspective; on one side, it acknowledges Islam as an integral component of national culture, while on the other, it may be perceived as a potential source of danger. Despite some ambiguity, the vast majority of Kazakhstani expresses support the state's religion policies.

What is the interplay between secularization and religiosity? Kazakhstan is a society that balances secularity and religiosity, with the populace preferring to follow secular ideas while religion plays an important role. Thus, demographic trends, particularly the increase in birth rates in certain parts of Kazakhstan after the turn of the twenty-first century, point to a return of traditionalism. Within this framework, it is difficult to disagree with R. Inglehart regarding the interpretation of societal secularization as a process characterized by non-linearity. In other words, if society is in crisis for an extended period, a person will lose a sense of security and confidence in the future, at which point the appeal to religion will gain increasing traction and scope.

The prevailing discourse surrounding “secularization vs. religiosity” is significantly influenced by the ontological framing of the Islamic tradition that has evolved within the Kazakh cultural context. This has developed a mindset characterized by tolerance and a reflective approach to religious culture, alongside the individual significance attributed to religiosity. Most Kazakhstanis feel that the revival of religion is simply the return of spirituality to the heart of human life, making it an essential component of a person's distinctive identity in a post-secular society. A study of Kazakhstan's religiosity reveals its ambiguity and adaptability. Kazakhstani society is secular, with faith as an essential condition for overcoming the vanity of life and establishing spirituality.

## Data Availability

The original contributions presented in the study are included in the article/supplementary material, further inquiries can be directed to the corresponding author.
