# Correction: Sociological study of religiosity in post-atheist Kazakhstan

**DOI:** 10.3389/fsoc.2026.1976992

**Published:** 2026-10-15

**Authors:** Aigul Zabirova, Natalya Seitakhmetova, Sholpan Zhandossova, Marhabbat Nurov

**Affiliations:** 1 Department of Public Opinion Monitoring, Kazakhstan Institute for Strategic Studies Under the President of the Republic of Kazakhstan, Astana, Kazakhstan; 2 Department of Religion Studies, Institute of Philosophy, Political Science and Religious Studies, Committee of Science, Ministry of Science and Higher Education of the Republic of Kazakhstan (CS MSHE RK), Almaty, Kazakhstan; 3 Faculty of Humanities and Law, Higher School of International Relations and Diplomacy, Turan University, Almaty, Kazakhstan

**Keywords:** religion, religiosity, secular, Kazakhstan, Muslims

In the published article, the conflict of interest lacked necessary details regarding a shared affiliation of authors Natalya Seitakhmetova and Sholpan Zhandossova, and reviewer Almasbek Shagyrbay. An investigation by the Research Integrity team found that this conflict of interest did not unduly impact the peer review process or scientific validity of the article.

The correct **Conflict of interest** statement appears below.

“The author(s) declared that this work was conducted in the absence of any commercial or financial relationships that could be construed as a potential conflict of interest.

The reviewer AS shares an affiliation with the authors NS and SZ.”

The original version of this article has been updated.

